# Infographics with Clinical Summaries Improve Medical Student Performance: A Within-Subject Intervention Study with Gender-Based Analysis

**DOI:** 10.1007/s40670-025-02384-x

**Published:** 2025-04-07

**Authors:** Luis Corral-Gudino, Clarisa Simón-Pérez, Jose Luis Pérez-Castrillón, Juan Carlos Martín-Escudero, Antonio Dueñas-Ruiz, Laisa Socorro Briongos-Figuero, Pablo Miramontes-González

**Affiliations:** 1 Department of Medicine, Dermatology and Toxicology, School of Medicine, University of Valladolid, Av. Ramón y Cajal, 7, , 47005 Valladolid, Spain; 2 Deparment of Surgery, Ophtalmology, Otolaryngology and Physiotherapy. School of Medicine, University of Valladolid, Av. Ramón y Cajal, 7, , 47005 Valladolid, Spain

**Keywords:** Medical education, undergraduate, Learning strategies, Visual learning, Infographics, Gender differences

## Abstract

**Introduction:**

This study aimed to evaluate the impact of infographics and social media threads (SMT) on academic performance in the Musculoskeletal Medical and Surgical Pathology (MMSP) course. Additionally, gender differences in the effectiveness of these tools were examined.

**Methods:**

A within-subject educational intervention study was conducted over three academic years, involving 459 fifth-year medical students. Infographics and SMT were exclusively provided for the rheumatology section of the MMSP course, while no additional tools were offered for the traumatology section, serving as a control. Students completed a final exam comprising multiple-choice questions (MCQs) for both sections. Survey data were collected to assess tool usage and perceptions (completed by 84.1%). Statistical analysis included Mann–Whitney *U*, Kruskal–Wallis, and Jonckheere-Terpstra tests with *Z*-score standardization.

**Results:**

The use of infographics significantly improved standardized scores in rheumatology MCQs (*p* = 0.035), particularly among male students (*p* = 0.046), while no impact was observed for traumatology section. SMT showed limited association with improved performance. Surveys indicated that 86% of students found infographics helpful for studying, and 85% would recommend them. In contrast, SMT received lower ratings, with 44% of students finding them useful for studying.

**Conclusions:**

Infographics effectively enhanced academic performance, with gender-specific variations in impact. In contrast, SMT had limited influence. These findings suggest that infographics are effective supplementary tools in medical education, especially when designed to align with diverse learning preferences. Further exploration of SMT’s potential and development of gender-inclusive teaching strategies is warranted.

**Graphical Abstract:**

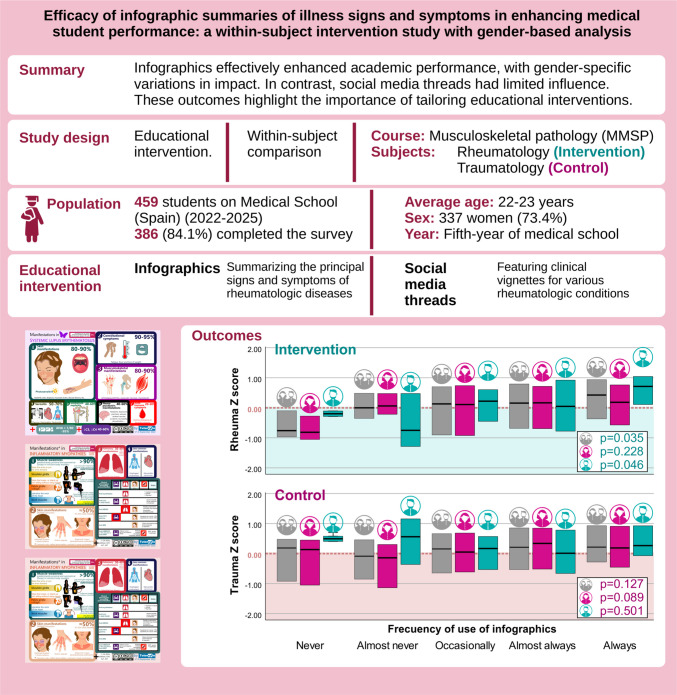

**Supplementary Information:**

The online version contains supplementary material available at 10.1007/s40670-025-02384-x.

## Introduction

Infographics (a visual representation of information that combines text, images, and design elements to enhance understanding and retention of complex concepts) have become increasingly popular as a tool in medical education, offering a visually engaging and concise way to present complex information [1]. There are several reasons why infographics are particularly effective in education. First, visual memory and information retention have been shown to be more robust than auditory memory, enabling learners to retain information more effectively [2, 3]. Furthermore, the integration of visual and verbal learning enhances comprehension and retention, as these modalities are processed and stored independently in long-term memory, in alignment with dual coding [4] and conjoint retention [5] theories. Graphical representations often outperform text alone when communicating complex concepts, as they allow learners to quickly grasp relationships and hierarchies [6]. Finally, presenting information visually reduces cognitive load, or the mental effort required to process and interpret information, making complex material more accessible and improving learning efficiency [7]. Additionally, infographics can foster active engagement by encouraging learners to interact with and interpret visual elements, thereby deepening understanding [8, 9]. A well-designed infographic effectively communicates a key idea or concept through visual elements, with minimal but purposeful text, thereby enhancing understanding and retention by simplifying complex information.

The versatility of infographics makes them suitable for diverse educational contexts, ranging from foundational knowledge to advanced problem-solving. In Table 1, various uses of infographics in medical education are presented [10–19].

**Table 1:** Application of infographics in medical education. The table summarizes various educational uses of infographics in medical training, including their underlying principles and supporting references

Applications of infographics in medical education	Underlying principle	Reference
To create educational materials that (a) Simplify complex concepts (b) Make medical information more accessible (c) Enhance learning and retention	Infographic combines visual and textual elements to provide succinct overviews of complex topics, making it easier for learners to grasp and retain information In addition, they can make information more accessible to students with different learning needs, including those with reading difficulties or language barriers	Gallagher SE, 2017 [10] Shanks JD, 2017 [11]
To create summaries of: (a) Lectures (b) Guidelines or protocols (c) Decision trees (d) Illness scripts, diagnostic schemas (e) Procedural skills (f) Quick reference visual aids for students during clinical rounds	Infographics can summarize lectures content, guidelines, protocols, or decision trees; can illustrate diseases sign or symptoms, processes, mechanisms, or treatments to create clinical vignettes; or can show step by step procedural skills like surgical techniques in a visually appealing manner In addition, infographics can be used as portable, visual guides to reduce stress and improve real-time decision-making in clinical rounds	Junhasavasdikul D, 2017 [12] Kathiah R, 2024 [13] Zimmermann AE, 2024 [14]
To facilitate exam preparation by: (a) Simplifying complex topics (b) Creating concept mapping (c) Defining quick reference guides (d) Highlighting key takeaways	Infographics can be used to outline effective exam preparation strategies, helping students organize their study efforts more effectively. They can help students to visualize relationships between different ideas and concepts They can serve as effective quick reference guides for students and summarize important information, making it easier for students to review key concepts before exams by allowing the students to focus on the most important information	Ying Hsiao P, 2019 [15]
To support clinical teaching as in (a) Classical lectures (b) Flipped classroom (c) Just-in-time teaching	Infographics can be used in flipped classroom settings to provide students with pre-class materials that prepare them for in-class discussions and activities	Hurtubise L, 2015 [16]
To facilitate student collaboration	The creation of an infographic could serve as a collaborative task, facilitating teamwork among peers to develop a well-designed and insightful project	Scott DA, 2020 [17] Navarrete-Muñoz EM, 2024 [19]
To encourage students peer teaching	Students can create infographics to teach their peers, reinforcing their own understanding while helping others	Sobhana NP, 2023 [18]

Despite their advantages and increasing incorporation into medical education strategies, the impact of infographics on measurable academic outcomes remains underexplored. A significant gap exists in the empirical evidence needed to substantiate their efficacy, particularly regarding their influence on academic performance in high-stakes assessments, such as multiple-choice question (MCQ) exams. While their potential utility is widely acknowledged [20, 21], and their effectiveness has been showed in non-medical academic fields as general education and other university disciplines [22]; few studies have rigorously evaluated whether their integration translates into meaningful improvements in learning outcomes specifically within the context of medical education. Bridging this gap is crucial to justify their continued use in medical curricula and to refine their design for optimal educational impact.

Alongside infographics, other multimedia tools such as social media threads may also enhance student performance [23, 24], and our study includes an exploratory evaluation of both formats.

This study seeks to evaluate the efficacy of infographics or social media threads in improving student performance on final MCQ exams in a medical school setting. To achieve this, we conducted a longitudinal analysis over three academic years, focusing on the course on *Musculoskeletal Medical and Surgical Pathology* (MMSP). The MMSP course is divided into two parts: the medical component, which corresponds to rheumatology, and the surgical component, which corresponds to orthopedics, with classes delivered in parallel. Infographics were exclusively developed for the rheumatology part, providing a unique opportunity to compare student performance across the two subject areas within the same cohort. All students completed a final MCQ exam covering both parts, allowing each student to serve as their own control in the analysis. This approach tries to minimize confounding factors and offers a robust framework to evaluate the true impact of infographics on learning outcomes.

In addition to evaluating the overall educational effectiveness of infographics, this study also examines potential gender-based differences in their impact. Previous research has suggested that educational tools may not have uniform impacts across demographic groups, and understanding such variations is essential for designing inclusive and effective learning resources.

The study focuses on these research questions: (1) Does using infographics or social media threads as an educational tool improve medical students'MCQ exam performance in the MMSP course? (2) Are there gender-based differences in the effectiveness of infographics or social media threads on student performance?

## Materials and Methods

### Study Design

**Fig. 1:**
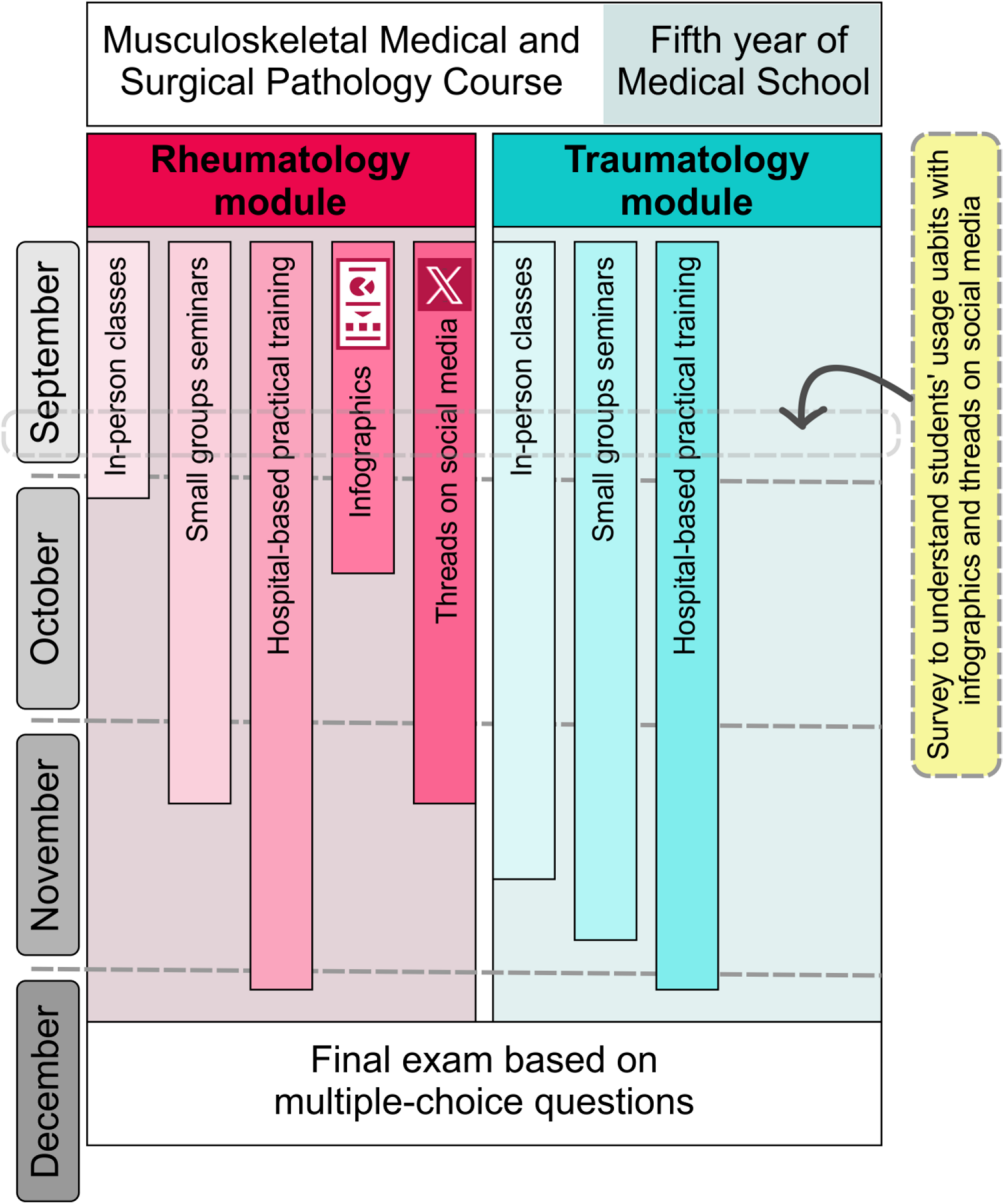
Study flow diagram

In this study, we used a within-subject educational intervention study that associates the use of infographics and social media threads with the results in a final MCQ exam. This study was conducted over three consecutive academic years (2022–2023, 2023–2024, 2024–2025), involving three cohorts of students enrolled in the MMSP course. The course is offered annually between September and December (Fig. 1).

The MMSP includes two main differentiate subjects: rheumatology and traumatology. All students were required to study both subjects as part of the course curriculum.

**Fig. 2:**
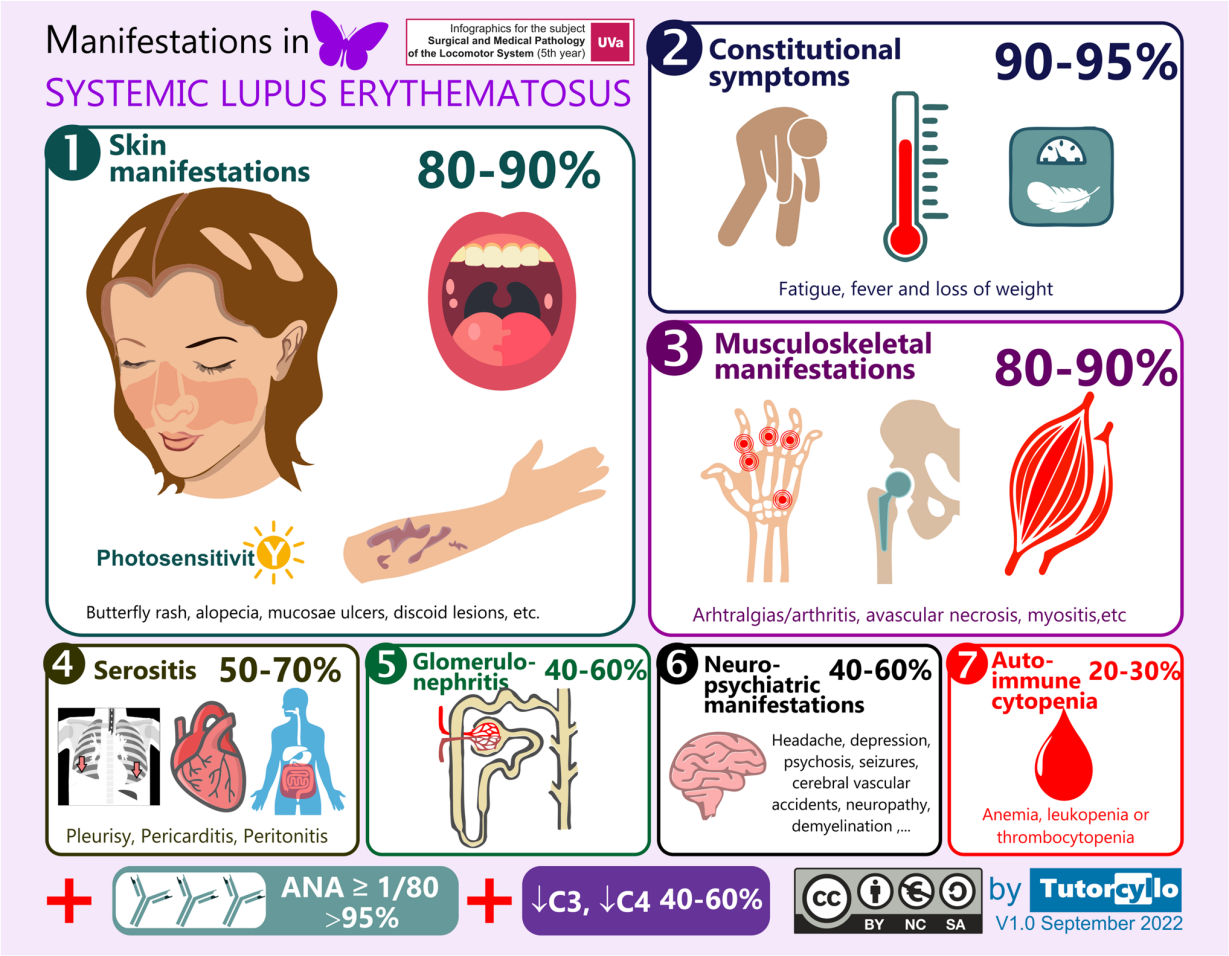
Example of infographic to summarize the main signs and symptoms of Systemic Lupus Erythematosus (SLE), organized by frequency. The infographic presents the organ systems commonly affected in SLE, accompanied by representative visual icons (e.g., malar erythema, oral ulcers, photosensitivity, alopecia for skin involvement). Each system is labeled with the approximate percentage of patients affected, based on published epidemiological data. The manifestations are arranged in descending order of frequency to provide a visual summary of the typical clinical spectrum of the disease

The study intervention was focused on the rheumatology and included two tools:

- Infographics: Most of the infographics created for this project were used as clinical summaries to illustrate the key features of rheumatologic diseases through clinical vignettes; additional infographics served as diagnostic schemas, guideline-based decision trees, or visual aids to reinforce key associations (e.g., tobacco use and rheumatoid arthritis), facilitating learning and exam preparation (see example in Fig. 2 and link to all the infographics in this interactive infographic link https://uvadoc.uva.es/handle/10324/68119). The infographics were made available to students as PDF files uploaded to the university’s online campus platform, integrated into classroom teaching sessions, archived in the institutional repository (https://uvadoc.uva.es/), and, in some cases, disseminated through social media platforms.
- Social media threads featuring clinical vignettes for various rheumatologic conditions (X/Twitter® during 2022–2023 and 2023–2024 or Instagram® during 2024–2025) (further information is available in Appendix 1).

The educational tools were provided to the students during the first half of the course.

In contrast, no additional educational tools, such as infographics or social media threads, were offered for the traumatology part of the course. This difference in educational support formed the basis for comparison.

To assess the frequency of use of infographics and social media threads by students and to know their perceptions of the interest and usefulness of these tools in facilitating their study of rheumatology, we conducted a survey at the end of September.

At the end of the semester, students completed a final exam comprising 100 MCQs: 25 questions on rheumatology and 75 on traumatology. Although the exam was administered simultaneously, scores for rheumatology and traumatology were calculated and graded independently.

### Ethical Approval

This project has been approved by the Institutional Review Board of the University of Valladolid.

### Setting and Participants

The study was conducted within the Medical Degree Program at the University of Valladolid, specifically during the fifth year of the program, which includes the MMSP course. The fifth year of medical school in Spain is the penultimate year of training and includes a range of clinical subjects with a strong practical focus, such as Preventive Medicine and Public Health, Radiology, Clinical Pharmacology, Geriatrics, Emergency Medicine, and Musculoskeletal Medical and Surgical Pathology, among others. All students enrolled in the MMSP course were invited to participate in the survey.

### Survey Characteristics

The survey consisted of the following questions:

#### Tool Evaluation

Students rated, on a scale from 1 (worst) to 5 (best), their assessment of five learning tools: (I) in-person lectures; (II) patient participation in lectures, where individuals shared their clinical histories with the students; (III) live-polls conducted in class using Ahaslides® or Wooclap®; (IV) infographics summarizing rheumatologic diseases; (V) social media threads featuring clinical vignettes.

Additional Likert questions assess (I) the frequency of use of infographics and social media threads; (II) whether infographics or social media threads aided their studying; (III) whether these tools increased their interest in the subject; (IV) whether they would recommend the use of these tools to future students.

#### Rationale for Selecting Within-Subject Educational Intervention Study Design

The selection of this study design was guided by the need to minimize bias when assessing the effectiveness of an educational intervention. Specifically, we aimed to avoid a scenario where the observed benefits of the intervention (infographics and social media threads) might be attributed to the characteristics of the students who chose to use them, such as higher motivation or greater academic ability, rather than the intrinsic value of the tools themselves.

To address this concern in our study, each student taking the rheumatology exam after the exposition to the infographics and social media threads serves as their own control in the traumatology exam where they are not being exposed to infographics or social media threads. Since all students were required to study both rheumatology and traumatology as part of the same course, this design allowed for a within-subject comparison of outcomes for the same individual under different conditions, reducing the likelihood that differences in outcomes were driven by individual student characteristics (e.g., differences in prior knowledge, motivation, or learning styles) rather than the intervention itself.

### Data Analysis

Survey data were collected through live polls conducted during class, while MCQ exam data were obtained from offline tests included in the Moodle platform. Individual responses on the use of infographics and social media were linked to each student’s final exam performance. This linkage enabled the within-subject analysis crucial to our study design. All data were managed confidentially in accordance with institutional data protection protocols and were used solely for research purposes.

Data were expressed as mean ± standard deviation (SD) for normally distributed variables or as median and interquartile range (IQR) for non-normally distributed variables, based on the results of the Kolmogorov–Smirnov test for normality.

Since most data series did not follow a normal distribution, non-parametric tests were employed to analyze differences. We used Mann–Whitney *U* test for independent samples to compare continuous variables differences between men and women. We used the Kruskal–Wallis test (KWt) to assess differences in categorical variables (e.g., exam performance based on the frequency of use of infographics or social media threads (divided in five ordinal categories: never, almost never, occasionally, almost always, and always)). When significant differences were identified (*p* < 0.005), the Jonckheere-Terpstra test (JTt) was performed to evaluate specific directional trends across the ordered categories. When the KWt was significant but there is no apparent trend in JTt, we assessed post hoc pairwise comparisons (Dunn’s test with Bonferroni correction) to identify the specific group differences.

Due to differences in the difficulty and grading scales of the exams in the rheumatology and traumatology MCQ exams during the three different academic courses, we decided to standardize the grades by using *Z*-scores. This method adjusts each grade based on the mean and standard deviation of its respective course, using the formula *Z* = (*X* − *μ*)/*σ*, where *X* is the grade, *μ* is the course mean, and *σ* is the standard deviation. *Z*-scores standardize grades to a scale with a mean of 0 and a standard deviation of 1, allowing for direct comparison between courses regardless of differences in grading scales or variability. This standardization allows for a fair comparison of student performance across the two subject areas, independent of inherent disparities in exam structure or scoring.

A significance level of 0.005 was set for all analyses, and data processing was conducted using SPSS version 21.

## Results

A total of 386 out of 459 (84.1%) fifth-year medical students who took the final examen of the MMSP course during 3 consecutive academic years completed the survey on the evaluation of infographics or the use of social media as a supplement to educational materials on the MMSP course. Students’ characteristics and the grades on a 10-point scale in the final MMSP exam is showed in Table 2.

**Table 2:** Students’ characteristics and grades on Musculoskeletal Medical and Surgical Pathology (MMSP) course

	Total (*n* = 459)	Women (*n* = 337)	Men (*n* = 122)	
Ethnicity European descent Latin American South Asian	454 3 2	334 1 2	118 2 0	-
Age 22–23 years Older	414 45	307 30	107 15	-
Rheumatology score Median (IQR)*	5.8 (3.0)	5.6 (3.2)	6.2 (2.6)	0.078**
KS	0.045	0.024	0.200	-
Traumatology score Median (IQR)*	7.6 (1.9)	7.5 (1.9)	7.6 (1.7)	0.646**
KS	< 0.001	0.002	0.008	-

*IQR* interquartile range, *KS* Kolmogorov–Smirnov test

^*^Grades on a 10-point scale

^**^Mann–Whitney *U* test

### Standardized Scores in Final MCQ Test Based on Infographic Use

**Fig. 3:**
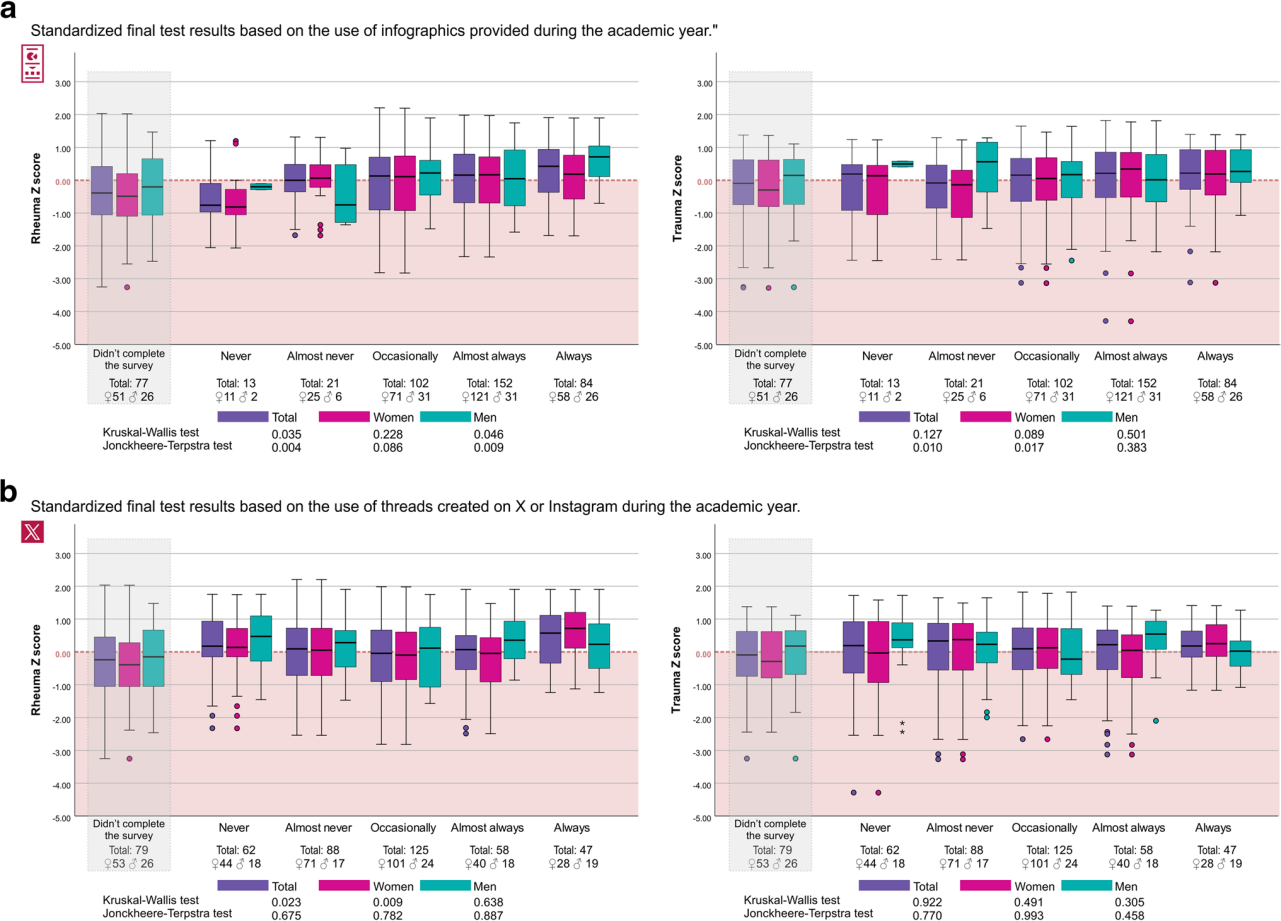
**a** Students’ standardized scores in the final Multiple Choice Question test for the Rheumatology and Traumatology sections of the MMSP course based on their use of infographics. **b** Students’ standardized scores in the final Multiple Choice Question test for the Rheumatology and Traumatology sections of the MMSP course based on their use of social media threads. These box plot diagrams display student scores in the final MCQ exam, grouped by frequency of infographic (**a**) or social media threads (**b**) use (never, almost never, occasionally, almost always, and always). The left side of each figure represents scores in the Rheumatology section, while the right side shows scores in the Traumatology section. Statistical results are shown at the bottom of each figure. The Kruskal–Wallis test was used to assess overall differences between groups, and the Jonckheere-Terpstra test was applied to evaluate the presence of a statistically significant trend in the data (i.e., whether higher infographic use was associated with improved performance)

Differences in adjusted scores based on *Z*-scores showed a significant association with the use of infographics in the final rheumatology exam according KWt (*p* = 0.035) with a specific trend across ordered categories according JTt (*p* = 0.004) (Fig. 3a). There were no differences in the traumatoloy exam (KWt *p* = 0.127). Pairwise comparisons revealed differences between the group that “never” used infographics and the groups that used them “almost always” (*p* = 0.032) and “always” (*p* = 0.006), as well as between the groups that used them occasionally and almost always (*p* = 0.039). However, these differences disappeared after applying the Bonferroni correction. (Data according to academic year are available in Appendixes 2 and 3.)

When the analysis was restricted to the group of women, no significant differences were observed for the adjusted scores in the rheumatology exam (*p* = 0.228) or the trauma exam (*p* = 0.089). Significant differences were found in the unadjusted scores for rheumatology between the group that “never” used infographics and those that used them “almost always” (*p* = 0.035) or “always” (*p* = 0.032).

In the group of men, a significant difference was found for the adjusted score in the rheumatology exam (*p* = 0.046) with specific trend across ordered categories according JTt (*p* = 0.009). There were no differences in the trauma exam (*p* = 0.501). In the unadjusted analysis, significant differences were observed between the groups that used infographics almost “never” and “always” (*p* = 0.015), as well as between the groups that used them “occasionally” and “always” (*p* = 0.036).

### Standardized Scores in Final MCQ Test Based on Social Media Threads Use

Differences in adjusted scores based on *Z*-scores showed a significant association with the use of social media in the final rheumatology exam according to KWt (*p* = 0.023) without a specific trend across ordered categories (JTt *p* = 0.675). The differences are restricted to the “always” groups comparisons with the other groups. There were no differences in the traumatology exam (*p* = 0.922) (Fig. 3b).

When the analysis was restricted to the group of women, the statistical significance indicating a general difference between the groups is maintained (KWt* p* = 0.009) without a specific trend across ordered categories (JTt *p* = 0.782). The differences again are restricted to the “always” groups comparisons with the other groups. There were no differences in the traumatology exam (*p* = 0.491). There were no differences according to social media use for both, rheumatology and traumatology exams in men (*p* = 0.638 and *p* = 0.305, respectively).

### Students’ Perspectives on Educational Tools Supplementing In-Person Classes

We asked our students about their assessment of the different medical tools used during the course (Table 3). The participation of real patients in class was the most highly rated educational resource (scored 4.8 out of 5), followed by the infographics (4.4) and in-person lectures (4.5). The use of threads on social media received the lowest rating (3.6).

**Table 3:** Students’ perspectives on educational tools supplementing in-person classes (comparison between women and men)

Rating on a Likert scale 1 to 5. Mean ± standard deviation	Total (*n* = 357)	Women (*n* = 265)	Men (*n* = 92)	*p*
Real patients in class	4.8 ± 0.6	4.8 ± 0.6	4.7 ± 0.7	0.216*
Use of infographics	4.4 ± 0.8	4.5 ± 0.8	4.3 ± 0.9	0.044*
In-person lectures	4.4 ± 0.9	4.4 ± 0.9	4.3 ± 0.9	0.186*
Live polls in class	4.0 ± 1.1	4.0 ± 1.1	3.9 ± 1.1	0.553*
Use of X/Twitter	3.6 ± 1.1	3.6 ± 1.1	3.6 ± 1.1	0.909*
Likert scale rating Total number (%)	(*n* = 386)	(*n* = 289)	(*n* = 97)	-
Infographic use Never Almost never Occasionally Almost always Always	13 (3%) 31 (8%) 102 (27%) 152 (40%) 84 (24%)	11 (4%) 25 (9%) 71 (25%) 121 (42%) 58 (20%)	2 (2%) 6 (6%) 31 (32%) 31 (32%) 26 (27%)	0.421**
Social media use* Never Almost never Occasionally Almost always Always	62 (16%) 88 (23%) 125 (33%) 58 (15%) 47 (12%)	44 (15%) 71 (25%) 101 (36%) 40 (14%) 28 (10%)	18 (19%) 17 (18%) 24 (25%) 18 (19%) 19 (19%)	0.081**
Aid studying (inf) Not at all A little Some Quite a lot Very much	2 (1%) 8 (2%) 46 (12%) 116 (31%) 207 (55%)	0 4 (1%) 35 (12%) 87 (31%) 156 (55%)	2 (2%) 4 (4%) 11 (11%) 29 (30%) 51 (53%)	0.075**
Aid studying (X) Not at all A little Some Quite a lot Very much	14 (4%) 63 (17%) 131 (35%) 109 (29%) 54 (15%)	10 (4%) 43 (16%) 104 (38%) 81 (29%) 38 (14%)	4 (4%) 20 (21%) 27 (28%) 28 (30%) 16 (17%)	0.976**
Increase interest (inf) Not at all A little Some Quite a lot Very much	4 (1%) 22 (6%) 76 (21%) 137 (37%) 131 (35%)	2 (1%) 15 (6%) 54 (20%) 101 (37%) 101 (37%)	2 (2%) 7 (7%) 22 (23%) 36 (37%) 30 (31%)	0.143**
Increase interest (X) Not at all A little Some Quite a lot Very much	23 (6%) 58 (16%) 128 (34%) 110 (29%) 56 (15%)	15 (5%) 45 (16%) 101 (36%) 85 (30%) 36 (13%)	8 (9%) 13 (14%) 27 (29%) 25 (27%) 20 (22%)	0.461**
Recommend (inf) No It is unlikely It is possible It is likely Without a doubt	5 (1%) 13 (3%) 41 (11%) 114 (30%) 212 (55%)	2 (1%) 8 (3%) 30 (10%) 82 (28%) 167 (58%)	3 (3%) 5 (5%) 12 (12%) 32 (33%) 45 (46%)	0.016**
Recommend (X) No It is unlikely It is possible It is likely Without a doubt	15 (4%) 35 (9%) 114 (31%) 121 (33%) 86 (23%)	10 (4%) 24 (9%) 88 (32%) 95 (34%) 59 (21%)	5 (5%) 11 (12%) 26 (27%) 26 (27%) 27 (28%)	0.945**

*Inf* infographics, *X* social media (2022–2023 and 2023–2024 X/Twitter, 2024–2025 Instagram)

^*^Mann–Whitney *U* test independent samples

^**^Kruskal–Wallis test

The infographics helped most students quite a lot or very much in studying the Rheumatology section of the course (86%) and significantly increasing their interest in the subject of Rheumatology quite a lot or very much (72%). They would recommend them to other students likely or without doubt in 85% of cases.

The infographics helped less than half of the students quite a lot or very much in studying the Rheumatology section of the course (44%) or increase their interest in the subject of Rheumatology quite a lot or very much in 44% of cases. The student would recommend them to other students likely or without doubt in 56% of cases.

## Discussion

Our study indicates that infographics as a complement to in-person classes are associated with improved MCQ test scores, particularly among male students. Conversely, the use of social networks showed no significant impact on student performance.

These findings align with existing literature in other disciplines where infographics have been linked to positive academic education [22]. However, studies in medical education remain scarce, with most focusing on perceptions rather than actual learning gains. Previous research has demonstrated that infographics enhance engagement and knowledge retention in biochemistry [25], public health courses [11], or Basic Life Support training [26]. Interactive infographics may yield better results than static ones [27].

Gender-based differences in infographic effectiveness suggest that male students benefit more, possibly due to their preference for multimodal learning approaches [28]. In contrast, female students often favor kinesthetic or aural engagement, which might explain the disparity [28, 29]. Studies suggest that male students show higher motivation towards infographic-based learning [30], but findings are not consistent across all educational interventions [31]. Despite the lack of significant exam score improvement among female students, their perception of infographics was highly positive. In fact, they rated the tool slightly higher than male students (mean rating 4.5 vs. 4.3), and 86% reported that infographics helped them study “quite a lot” or “very much.” These findings indicate that infographics may provide educational value beyond measurable academic performance, enhancing engagement and accommodating a range of learning preferences.

Medical students in our study perceived infographics as valuable study tools, aligning with previous research [19, 25, 32–34]. Some students even preferred infographics over traditional teaching methods [35]. However, social media threads received lower ratings despite the widespread use of social media among students. This could be due to the perception of social platforms as primarily for leisure, creating a disconnect when used for educational purposes [36]. Additionally, social media content created by professors may lack the peer-driven dynamic students prefer. Even after transitioning from X to Instagram in the course 2024–2025 to better align with our student habits [37], no significant change in engagement was observed, highlighting the challenge of leveraging social media for academic purposes.

The highest-rated educational tool among students was interaction with real patients, emphasizing the importance of practical experience in medical education.

Several limitations should be considered in interpreting our findings. Firstly, the lack of randomization introduces potential selection bias, as students with stronger study habits may have been more inclined to use infographics and social media resources. To mitigate this, we compared performance between two parallel course sections, Rheumatology (with intervention) and Traumatology (without intervention). The specific association of infographics with improved performance in Rheumatology supports their potential efficacy. Randomization was not feasible in certain educational contexts where curricular decisions were implemented uniformly across entire course sections. However, the within-subject design partially mitigates this limitation by controlling interindividual variability. Future research could strengthen the evidence by conducting randomized controlled trials in environments where random assignment is possible, such as during elective modules or in blended learning settings. Secondly, 15.9% of students did not complete the survey, potentially introducing nonresponse bias, but the high participation rate of 84.1% helps maintain study validity, although we did not analyze the characteristics of non-respondents, which could have introduced unmeasured differences. Future studies should consider incorporating follow-up strategies (e.g., reminders, brief surveys, or incentives) to enhance response rates and explore whether non-respondents differ systematically from participants. Thirdly, context dependency is another limitation, as the intervention was tailored to clinical subjects and may not apply to basic sciences with different teaching methods. Clinical teaching incorporates problem-solving and applied knowledge, which may affect how visual tools like infographics are utilized. Future research should investigate the effectiveness of infographics in basic science courses (e.g., biochemistry, anatomy) and in specialties that predominantly use theoretical instruction, to determine whether the advantages apply to different content types and educational methods. Single-institution design may also limit generalizability, although infographics are adaptable across curricula. Cognitive load theory and multimedia learning principles support the effectiveness of infographics, suggesting broader applicability. Replication of this study in different medical schools would help assess the external validity and generalizability of our findings. Multicenter studies or cross-institutional collaborations could provide richer insights into the effectiveness of visual learning tools in diverse learning environments. Fourthly, confounding variables such as prior academic performance and other supplementary resources were not fully accounted for in the study design. These factors may have impacted both the adoption of infographics and the results of examinations. Future research should strive to identify and control these variables through baseline assessments, learner profiling, or mixed-methods approaches, in order to more accurately determine the specific effect of educational interventions. Additionally, the reliance on MCQ as the sole outcome measure may not capture deeper comprehension or clinical application skills. Despite these limitations, the robust sample size and objective performance data add strength to our conclusions. Future studies could complement MCQ-based assessments with alternative evaluation tools such as Objective Structured Clinical Examinations (OSCEs), case-based discussions, problem-solving tasks, or even long-term retention assessments to evaluate the impact of infographics on clinical reasoning, application of knowledge, and critical thinking.

Future research should explore digital tools’ effectiveness across different educational settings and investigate the potential of artificial intelligence (AI) in creating personalized learning experiences. AI technologies could create customized infographics and social media content for students, adjusting complexity and style based on learning needs. However, these are just a small part of the wide array of resources that AI can generate. AI can also develop adaptive educational tools such as virtual patient simulations, interactive tutors, or chat-based learning assistants, enhancing engagement and learning outcomes through personalized and scalable solutions in medical education.

In summary, our study confirms that infographics can enhance academic performance, particularly for male students, emphasizing the need to consider individual learning preferences in educational strategies. The limited impact of social media threads highlights the challenges of integrating social platforms into academic environments. While these findings support the value of tailored visual tools in medical education, they should be interpreted considering certain limitations as the single-institution design and the reliance on MCQ exams as the sole measure of learning. Future studies in diverse educational settings and with broader assessment tools are needed to further validate and expand upon these results.

## Supplementary Information

Below is the link to the electronic supplementary material.

Supplementary file1 (DOCX 1040 KB)

## Data Availability

The data that support the findings of this study are available from the corresponding author (LCG), upon reasonable request.

## Glossary

Infographics: Visual representations that combine text and images to present information in a concise, engaging, and easily digestible format, often used as a learning aid
Social media threads (SMT): A series of connected posts on social media platforms (e.g., X, Instagram) used to share information or discuss a topic in a sequential manner.
Musculoskeletal Medical and Surgical Pathology (MMSP): A medical course covering both the medical (rheumatology) and surgical (traumatology) aspects of musculoskeletal conditions.
Cognitive load: The mental effort required to process and understand information, which can impact learning outcomes.
Dual coding theory: A theory suggesting that combining visual and verbal information enhances memory and learning by engaging different cognitive processes.
Visual learning: A learning style where individuals prefer to use images, diagrams, and visual aids to understand and retain information
Within-subject educational intervention: A study design where the same participants are exposed to multiple conditions, serving as their own control to reduce variability.
Gender differences: Variations in behavior, preferences, or outcomes based on gender, which can influence the effectiveness of educational tools.
