# Supplementary material for: Infographics with Clinical Summaries Improve Medical Student Performance: A Within-Subject Intervention Study with Gender-Based Analysis
Source: Med Sci Educ. 2025 Apr 7;35(4):1947–58. doi: 10.1007/s40670-025-02384-x (PMC12532518; doi:10.1007/s40670-025-02384-x)
Supplement: Supplementary file 1 — Supplementary file1 (DOCX 1040 KB) [file 40670_2025_2384_MOESM1_ESM.docx]

**Supplementary materials**

**Appendix 1: Threads on social media**

| **Title** | **Number of Tweets or Instagram images** | **Date** | **Link** |
| --- | --- | --- | --- |
| Subject Presentation: Locomotor System | 10 | 4 Sept 2022 | <https://twitter.com/Luis_Corral_Gud/status/1566508156955525122?s=20&t=qJendbhO_B8tC8hTQCFBkw> |
| Introduction to Rheumatoid Arthritis | 5 | 5 Sept 2022 | <https://twitter.com/Luis_Corral_Gud/status/1566828367244050436?s=20&t=vJXobbOBgijvJ2vrXj4rmA> |
| Rheumatoid Arthritis* | 6 | 6 Sept 2022 | <https://twitter.com/Luis_Corral_Gud/status/1567051962931093504?s=20&t=NIptXbODM7ATzZlUu5bL3g> |
| Infographic: Lupus | 2 | 12 Sept 2022 | <https://twitter.com/Luis_Corral_Gud/status/1569224803508166656?s=20&t=vyHNZ7pbReDSu34f1hMFqA> |
| Infographic: Scleroderma | 1 | 13 Sept 2022 | <https://twitter.com/Luis_Corral_Gud/status/1569581808345309185?s=20> |
| Infographic: Vasculitis | 9 | 14 Sept 2022 | <https://twitter.com/Luis_Corral_Gud/status/1569950208536252416?s=20> |
| Infographic: Inflammatory miopathies | 7 | 19 Sept 2022 | <https://twitter.com/Luis_Corral_Gud/status/1571764140988895235?s=20> |
| Polyarthritis | 8 | 9 Sept 2022 | <https://twitter.com/Luis_Corral_Gud/status/1568140306385670144?s=20&t=EH3PVFEOs-wUx8tc3ag5uw> |
| Osteoporosis | 6 | 23 Sept 2022 | <https://twitter.com/Luis_Corral_Gud/status/1573210801216913409?s=20> |
| Presentation of the Map: Semiology in Image | 5 | 9 Nov 2022 | <https://twitter.com/Luis_Corral_Gud/status/1590383005520269312?t=I4t-fFzdSI79ZL4HLNiUGA&s=09> |
| RS3PE syndrome** | 2 | 5 Sept 2023 | <https://x.com/Luis_Corral_Gud/status/1698960828869824570> |
| Diagnostic Algorithms | 8 | 7 Sept 2023 | <https://x.com/Luis_Corral_Gud/status/1699649348408050029> |
| Sjögren's Disease | 6 | 12 Sept 2023 | <https://x.com/Luis_Corral_Gud/status/1701502580298252733> |
| Infographic: Behçet's Disease | 1 | 13 Sept 2023 | <https://x.com/Luis_Corral_Gud/status/1701821653473243321> |
| Infographic: VEXAS Syndrome | 5 | 14 Sept 2023 | <https://x.com/Luis_Corral_Gud/status/1702223510049870225> |
| Instagram Profile: Semiology in Images | 1 | 14 March 2024 | <https://www.instagram.com/p/C4gW5DeC7_T/?utm_source=ig_web_copy_link&igsh=MzRlODBiNWFlZA==> |
| Autoimmune systematic diseases educational videos | 1 | 22 June 2024 | <https://www.instagram.com/reel/C8g9Nc4CB9b/?utm_source=ig_web_copy_link&igsh=MzRlODBiNWFlZA==> |
| Smoke and rheumatoid arthritis*** | 1 | 4 Sept 2024 | <https://www.instagram.com/p/C_fIvX2CD0N/?utm_source=ig_web_copy_link&igsh=MzRlODBiNWFlZA==> |
| Antiphospholipid syndrome | 4 | 9 Sept 2024 | <https://www.instagram.com/p/C_s-xhONwm2/?utm_source=ig_web_copy_link&igsh=MzRlODBiNWFlZA==> |
| Sjögren syndrome | 6 | 11 Sept 2024 | <https://www.instagram.com/p/C_xK40gtOcb/?utm_source=ig_web_copy_link&igsh=MzRlODBiNWFlZA==> |
| Behçet syndrome | 8 | 15 Sept 2024 | <https://www.instagram.com/p/C_7ect8tRGj/?utm_source=ig_web_copy_link&igsh=MzRlODBiNWFlZA==> |
| Antiphospholipid syndrome | 5 | 15 Sept 2024 | <https://www.instagram.com/p/C_7fknKNgL7/?utm_source=ig_web_copy_link&igsh=MzRlODBiNWFlZA==> |
| Real patients in in-person classes | 1 | 19 Sept 2024 | <https://www.instagram.com/p/DAGYUf2NhfG/?utm_source=ig_web_copy_link&igsh=MzRlODBiNWFlZA==> |

*Example of a twitter thread (5 September 2022)

| 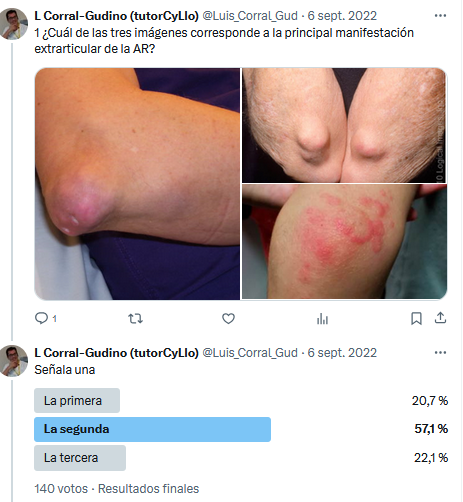 | 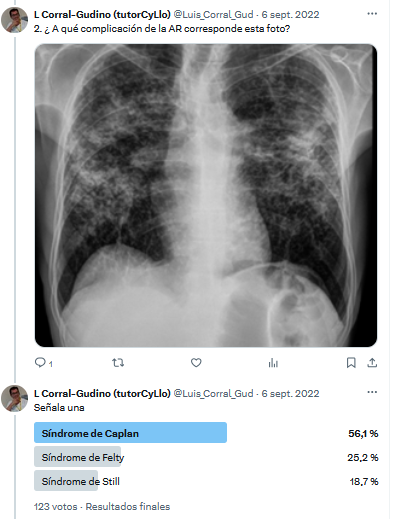 |
| --- | --- |

**Example of a twitter thread (5 September 2023)

| 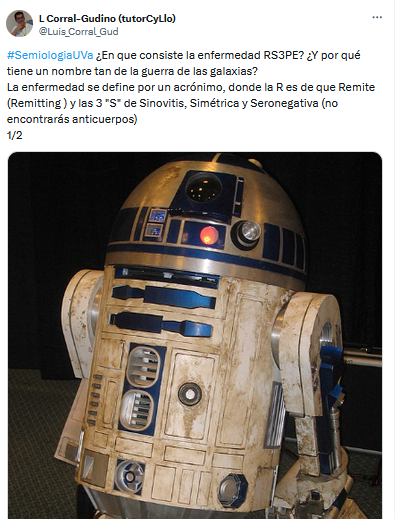 | 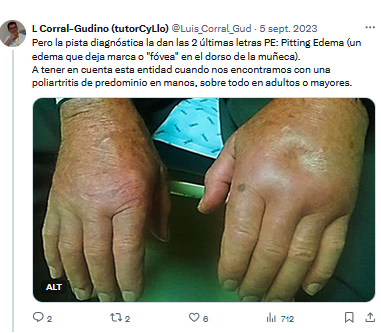 |
| --- | --- |

*** Example of an Instagram post (4 September 2024)


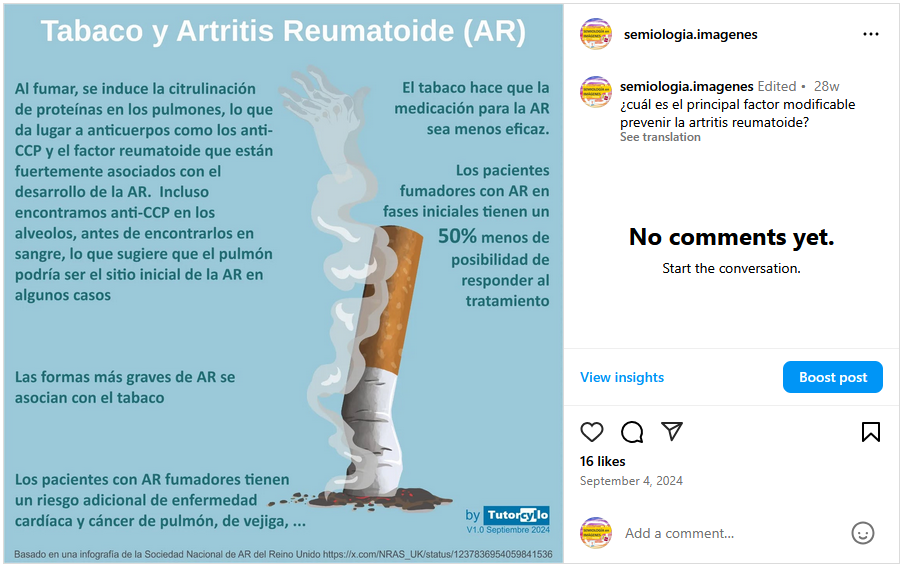


**Appendix 2: Students marks according to academic year.**

|  | Autoimmune diseases and rheumatology | | | | Traumatology | | | |
| --- | --- | --- | --- | --- | --- | --- | --- | --- |
| Curse | 2022-23 | 2023-24 | 2024-25 | p* | 2022-23 | 2023-24 | 2024-25 | p* |
| Median (IQR) | 6.5 (2.9) | 5.6 (3.2) | 5.3 (2.5) | <0.001 | 7.6 (2.2) | 7.7 (1.8) | 7.4 (1.9) | 0.096 |
| Mean ± SD | 6.5 ± 2.0 | 5.6 ± 1.9 | 5.1 ± 1.8 | - | 7.3 ± 1.6 | 7.5 ± 1.3 | 7.2 ± 1.4 | - |
| KS | 0.014 | 0.014 | 0.200 | - | <0.001 | .060 | .004 | - |
| Women /men | 110/47 | 115/31 | 112/44 | - | 110/47 | 115/31 | 112/44 | - |

*Kruskal-Wallis for independent samples

KS: Kolmogorov-Smirnov

**Appendix 3: Students use infographics and social media according to academic year.**

|  | 2022-23 | 2023-24 | 2024-2024 |
| --- | --- | --- | --- |
| Infographic use  Never  Almost never  Occasionally  Almost always  Always  Not answer | 7 (5%)  8 (5%)  36 (23%)  44 (28%)  32 (20%)  30 (19%) | 4 (3%)  8 (6%)  37 (25%)  44 (30%)  24 (16%)  29 (29%) | 2 (1%)  15 (10%)  29 (19%)  64 (41%)  28 (18%)  18 (12%) |
| X/Instagram* use  Never  Almost never  Occasionally  Almost always  Always  Not answer | X  26 (17%)  21 (13%)  32 (21%)  20 (13%)  24 (15%)  33 (21%) | X  23 (16%)  24 (16%)  41 (28%)  19 (13%)  12 (8%)  27 (19%) | Instagram  13 (8%)  43 (28%)  51 (33%)  19 (12%)  11 (7%)  19 (12%) |
